# Comparison of microbiological diagnosis of urinary tract infection in young children by routine health service laboratories and a research laboratory: Diagnostic cohort study

**DOI:** 10.1371/journal.pone.0171113

**Published:** 2017-02-15

**Authors:** Kate Birnie, Alastair D. Hay, Mandy Wootton, Robin Howe, Alasdair MacGowan, Penny Whiting, Michael Lawton, Brendan Delaney, Harriet Downing, Jan Dudley, William Hollingworth, Catherine Lisles, Paul Little, Kathryn O’Brien, Timothy Pickles, Kate Rumsby, Emma Thomas-Jones, Judith Van der Voort, Cherry-Ann Waldron, Kim Harman, Kerenza Hood, Christopher C. Butler, Jonathan A. C. Sterne

**Affiliations:** 1 School of Social and Community Medicine, University of Bristol, Bristol, United Kingdom; 2 Centre for Academic Primary Care, NIHR School of Primary Care Research, School of Social and Community Medicine, University of Bristol, Bristol, United Kingdom; 3 Specialist Antimicrobial Chemotherapy Unit, Public Health Wales Microbiology Cardiff, University Hospital Wales, Cardiff, United Kingdom; 4 North Bristol NHS Trust, Bristol, United Kingdom; 5 Kleijnen Systematic Reviews Ltd, York, United Kingdom; 6 NIHR Biomedical Research Centre at Guy’s and St Thomas’ NHS Foundation Trust and King’s College London, Department of Primary Care and Public Health Sciences, London, United Kingdom; 7 Bristol Royal Hospital for Children, University Hospitals Bristol, NHS Foundation Trust, Bristol, United Kingdom; 8 South East Wales Trials Unit (SEWTU), Institute for Translation, Innovation, Methodology and Engagement, School of Medicine, Cardiff University, Cardiff, United Kingdom; 9 Primary Care and Population Sciences Division, University of Southampton, Southampton, United Kingdom; 10 Cochrane Institute of Primary Care & Public Health, School of Medicine, Cardiff University, Cardiff, United Kingdom; 11 Department of Paediatrics and Child Health, University Hospital of Wales, Cardiff, United Kingdom; 12 Nuffield Department of Primary Care Health Sciences, University of Oxford, Oxford, United Kingdom; Universite Paris Descartes, FRANCE

## Abstract

**Objectives:**

To compare the validity of diagnosis of urinary tract infection (UTI) through urine culture between samples processed in routine health service laboratories and those processed in a research laboratory.

**Population and methods:**

We conducted a prospective diagnostic cohort study in 4808 acutely ill children aged <5 years attending UK primary health care. UTI, defined as pure/predominant growth ≥10^5^ CFU/mL of a uropathogen (the reference standard), was diagnosed at routine health service laboratories and a central research laboratory by culture of urine samples. We calculated areas under the receiver-operator curve (AUC) for UTI predicted by pre-specified symptoms, signs and dipstick test results (the “index test”), separately according to whether samples were obtained by clean catch or nappy (diaper) pads.

**Results:**

251 (5.2%) and 88 (1.8%) children were classified as UTI positive by health service and research laboratories respectively. Agreement between laboratories was moderate (kappa = 0.36; 95% confidence interval [CI] 0.29, 0.43), and better for clean catch (0.54; 0.45, 0.63) than nappy pad samples (0.20; 0.12, 0.28). In clean catch samples, the AUC was lower for health service laboratories (AUC = 0.75; 95% CI 0.69, 0.80) than the research laboratory (0.86; 0.79, 0.92). Values of AUC were lower in nappy pad samples (0.65 [0.61, 0.70] and 0.79 [0.70, 0.88] for health service and research laboratory positivity, respectively) than clean catch samples.

**Conclusions:**

The agreement of microbiological diagnosis of UTI comparing routine health service laboratories with a research laboratory was moderate for clean catch samples and poor for nappy pad samples and reliability is lower for nappy pad than for clean catch samples. Positive results from the research laboratory appear more likely to reflect real UTIs than those from routine health service laboratories, many of which (particularly from nappy pad samples) could be due to contamination. Health service laboratories should consider adopting procedures used in the research laboratory for paediatric urine samples. Primary care clinicians should try to obtain clean catch samples, even in very young children.

## Introduction

Urinary tract infection (UTI) affects 6% of acutely unwell children presenting to UK general practice.[1] Timely diagnosis and treatment may alleviate short-term symptoms and could potentially prevent long-term adverse consequences such as renal scarring, impaired renal growth, recurrent pyelonephritis, impaired glomerular filtration, hypertension, end stage renal disease, and pre-eclampsia.[2–4] However establishing a diagnosis in pre- or early-school aged children is challenging; many are pre-verbal and collection of uncontaminated urine samples is difficult.[5] UK National Institute for Health and Clinical Excellence (NICE) guidelines say that a “clean catch” sample is the recommended method for urine collection, but urine collection pads are advised if this is not possible.[6] The American Academy of Pediatrics practice clinical guidelines recommend that urine is collected by catheterization or suprapubic aspiration in young children [7], but these collection methods are invasive and may be unacceptable to parents, and so are uncommon in UK primary care.

Laboratory diagnosis is based on colony counts following culture. UTI is typically caused by a single organism present in high concentration, usually ≥10^5^ colony-forming units (CFU) per mL.[8] Laboratory guidelines differ regarding the extent of growth required to confirm UTI.[9, 10] NICE guidelines do not provide a definitive threshold.[6] National Health Service (NHS) laboratories are the routine health service laboratories in the UK and they follow the UK Standards for Microbiological Investigation [10] for examination of urine, but application varies between laboratories.

The aim of this study was to compare the validity of diagnosis of UTI through urine culture between samples processed in routine health service laboratories and those processed in a research laboratory, using data from a diagnostic cohort study among unselected children aged <5 years presenting to primary care in England and Wales with acute illnesses. Because there is no independent reference (“gold-standard”) test for diagnosis of UTI, we could not directly assess the diagnostic accuracy of culture results. We therefore evaluated the validity of diagnosis by examining associations of pre-specified parent-reported symptoms, clinician-reported signs, and urine dipstick test results with urine culture positivity in the two types of laboratory.

## Population and methods

The Diagnosis of Urinary Tract infection in Young children (DUTY) study was a multicentre, prospective, diagnostic cohort study. The methods of recruitment are described in detail in the study protocol.[11] Children were eligible if they were aged <5 years, presented to primary care with any acute illness episode of <28 days duration and had constitutional or urinary symptoms associated with their acute illness. Children were excluded if they were not constitutionally unwell, had a neurogenic or surgically reconstructed bladder, used a urinary catheter, presented with trauma, or had taken antibiotics within the past week. We recruited participants from 233 primary care sites (225 General Practitioner [GP] practices, four Walk-in Centres and four paediatric Emergency Departments) across England and Wales between April 2010 and April 2012. Clinicians were asked to recruit consecutive eligible children. Following written informed parental consent, data were collected on personal details, medical history, presenting symptoms and results of the clinical examination including a clinician-reported global impression of illness severity (score 0–10). Multi-centre ethical approval was granted for this study by the South West Southmead Research Ethics Committee, Ref #09/H0102/64.

Urine samples were obtained by clean catch where possible, for children who were toilet trained or for whom the parent/guardian was happy to attempt such collection. ‘Newcastle Nappy Pads’ were used for children still using nappies (diapers) whose parent/guardian did not think clean catch would be successful. The Research Nurse, wearing disposable gloves, removed the pad and squeezed the urine into a sterile bowl. Samples were dipstick tested for blood, protein, glucose, ketones, nitrite, leukocyte esterase, pH and specific gravity using Siemens/Bayer Multistix 8SG.

Urine samples were split into two fractions with the priority fraction sent to the routine health service laboratory usually used by the recruiting site. The samples were sent to the local routine heath service laboratory using the site’s normal method of transport. All samples were sent to the laboratory as soon as possible after collection, but were refrigerated if transport to the laboratory was delayed for more than four hours. If sufficient urine was available, a second fraction was sent to the Specialist Antimicrobial Chemotherapy Unit, Public Health Wales Microbiology Laboratory (“Research Laboratory”; RL). Samples were sent to routine heath service laboratories using sterile urine containers, and the research laboratory via Royal Mail Safeboxes^TM^ in a urine Monovette containing boric acid. Health Service laboratories used local Standard Operating Procedures (SOPs) and reported bacterial growth (<10^3^; 10^3^-<10^5^; or ≥10^5^ CFU/mL), purity (pure/predominant; mixed growth 2 species; mixed growth >2 species), speciation for up to two species, and microscopy for white and red cells. All local health service laboratories were ‘Clinical Practice Accredited’ and NHS laboratory SOPs were used to process DUTY urine samples. All local health service laboratory SOPs were based on the Public Health England guideline for the investigation of urine.[10] A summary of these processes is given in S1 Table. In the research laboratory, automated microscopy was performed using the IRIS IQ200 Sprint (Instrumentation Laboratories) then 50μL cultured onto chromogenic agar and Columbia blood agar using a spiral plater (Don Whitley, UK) (S2 Table). Absolute colony counts (range 10^1^−10^10^ CFU/mL) were recorded for all organisms present, and species identification (using a Phoenix automated ID/AST system [BD diagnostics] plus conventional methods) for organisms present at ≥10^3^ CFU/mL. Sensitivities to first line antimicrobials were recorded for pure/predominant cultures and the presence of antimicrobial substances investigated by inhibition of growth of Bacillus subtilis (NCTC 10400).

Analyses were restricted to samples with results from both health service and research laboratories. Uropathogens were defined as members of the *Enterobacteriaceae* family. The reference standard was UTI, defined according to UK guidelines [6] as pure/predominant growth ≥10^5^ CFU/mL of a uropathogen. For health service laboratories, samples with pure/predominant growth of a uropathogen at ≥10^5^ CFU/mL were considered UTI positive. For the research laboratory, samples with growth of ≥10^5^ CFU/mL of a single uropathogen (“pure growth”) or growth of ≥10^5^ CFU/mL of a uropathogen with ≥3 log_10_ difference between growth this and the next species (“predominant growth”) were considered UTI positive. Agreement was assessed using kappa statistics. Because we found that agreement between health service and research laboratories was better for samples collected through clean catch than for those collected using nappy pads, most analyses were stratified by urine collection method. Analyses were additionally stratified by urine collection method and age (0 to <2, 2 to <3 and 3 to <5 years). Further details of study methods are provided in S1 Text.

*A priori*, we selected the “index test” for this study to be a small number of symptoms, signs and dipstick test results reported in the literature to be clearly related to UTI [12]: urinary symptoms (pain/crying when passing urine, passing urine more often, changes in urine appearance); temperature ≥39°C, and nitrite or leukocyte positive results from urine dipstick tests. We decided *a priori* (based only on inspection of symptom frequencies) to dichotomise symptom response categories as “no, slight or not known” and “moderate or severe”. Observers assessing the “index test” differed from and were blind to the reference standard (and vice versa).

We used multivariable logistic regression, including the selected symptoms, signs and dipstick test results, to quantify associations with UTI positivity. From the logistic regression equation, we plotted Receiver Operating Characteristic (ROC) curves and used the area under the ROC curve (AUC) with 95% confidence interval (CI) to quantify diagnostic accuracy. The maximum value of the AUC is 1 (perfect prediction) while a value of 0.5 corresponds to no association with any predictor. We estimated AUCs: (1) stratifying by age (<3 and ≥3 years), (2) allowing for “not known” categories for variables for which these occurred sufficiently frequently, (3) stratifying by whether samples were collected at the surgery or at home, (4) stratifying by time between taking urine sample and laboratory sample receipt (<24 hours and ≥24 hours), (5) stratifying routine heath service laboratory results according to extent of pure/predominant growth (≥10^5^, ≥10^3^-<10^5^ CFU/mL), (6) stratifying research laboratory results according to extent of pure/predominant growth (≥10^7^, ≥10^6^-<10^7^, ≥10^5^-<10^6^, ≥10^4^-<10^5^, ≥10^3^-<10^4^ CFU/mL), (7) stratifying according to whether white blood cell count was <30 or ≥30/mm^3^ and (8) stratifying research laboratory results according to whether growth was pure or predominant. Analyses were carried out using Stata^TM^ version 12.

## Results

Of 7163 children recruited to the study, 4828 had results from both laboratories and 4808 had information available on candidate predictors (S1 Fig). The children who were included in this study were older (mean age 29 months) compared to children who were recruited to DUTY, but were not included in this study (mean age 21 months). There were no gender differences (49.0% vs 49.6% male, for those included and not included in our study, respectively), but there was a small difference in ethnicity (83.3% white in our study vs 80.3% white in those who were recruited but not included). Most children who were included in the study (4543, 94.5%) were recruited from GP surgeries (Table 1). There were approximately equal numbers of boys and girls. A total of 2884 children (60%) were aged <3 years and 140 children (2.9%) were aged <3 months. Urine samples were collected using clean catch for 758 (26.3%) of 2884 children aged <3 years and 1861 (96.7%) of 1924 children aged 3–5 years. Among children aged <3 years, samples were obtained in the surgery in 1470 (51.0%) children aged <3 years and 1477 (76.8%) aged 3–5 years. 94% of samples were provided within 24 hours of clinical examinations. Health service laboratory transport systems were faster than the research laboratory with 72.3% vs. 29.6% samples arriving in the laboratory within 24 hours. Parents reported the following symptoms in their children as a moderate or severe problem: pain or crying when passing urine 217 (4.5%), passing urine more often 484 (10.1%), day or bed wetting when previously dry 209 (4.3%) and change in urine appearance 523 (10.9%). A history of UTI was reported in 221 (4.6%) children, 140 of whom were aged ≥3 years. 185 (3.8%) children had a temperature ≥39°C, and fever at any time during the illness was a moderate/severe problem in 2581 (53.7%) participants. Both nitrite (12.9% compared with 2.2%) and leukocyte (16.0% compared with 10.8%) urine dipstick positivity were more common in children aged <3 than ≥3 years. We are not aware of any adverse events resulting from the measurement of “index” or reference tests.

**Table 1:** Characteristics of children and urine samples collected via clean catch or nappy pads, for the 4808 children with both a routine health service laboratory and research laboratory result.

Variable	Category	Age <3 years	Age 3–5 years
Gender	Male	1439 (49.9%)	919 (47.8%)
	Female	1445 (50.1%)	1005 (52.2%)
Age (years)	0 to <1	1016 (35.2%)	0
	1 to <2	942 (32.7%)	0
	2 to <3	926 (32.1%)	0
	3 to <4	0	1099 (57.1%)
	4 to <5	0	825 (42.9%)
Ethnicity	White	2429 (84.2%)	1575 (81.9%)
	Non-white	431 (14.9%)	328 (17.1%)
	Not known	24 (0.8%)	21 (1.1%)
Recruitment site	GP surgery	2716 (94.2%)	1827 (95.0%)
	Emergency department	128 (4.4%)	66 (3.4%)
	Walk in centre	40 (1.4%)	31 (1.6%)
Sample method	Clean catch	758 (26.3%)	1861 (96.7%)
	Nappy pad	2126 (73.7%)	63 (3.3%)
Location of sample collection	Surgery	1470 (51.0%)	1477 (76.8%)
	Home	1414 (49.0%)	447 (23.2%)
Time between clinical exam and taking urine sample	< 24 hours	2683 (93.0%)	1853 (96.3%)
	≥24 hours	201 (7.0%)	71 (3.7%)
Time between taking urine sample and laboratory sample receipt	Health service laboratory < 24 hours	2045 (70.9%)	1432 (74.4%)
	Health service laboratory ≥ 24 hours	839 (29.1%)	492 (25.6%)
	Research laboratory < 24 hours	816 (28.3%)	608 (31.6%)
	Research laboratory ≥ 24 hours	2068 (71.7%)	1316 (68.4%)
Pain/crying when passing urine	No or slight problem	1812 (62.8%)	1734 (90.1%)
	Moderate or severe problem	92 (3.2%)	125 (6.5%)
	Not known	980 (34.0%)	65 (3.4%)
Passing urine more often	No or slight problem	1618 (56.1%)	1604 (83.4%)
	Moderate or severe problem	228 (7.9%)	256 (13.3%)
	Not known	1038 (40.0%)	64 (3.3%)
Changes in urine appearance	No or slight problem	2206 (76.5%)	1539 (80.0%)
	Moderate or severe problem	297 (10.3%)	226 (11.8%)
	Not known	381 (13.2%)	159 (8.3%)
Day or bed wetting when previously dry	No or slight problem	364 (12.6%)	1551 (80.6%)
	Moderate or severe problem	45 (1.6%)	164 (8.5%)
	Wears nappies day and night	2377 (82.4%)	70 (3.6%)
	Not known	98 (3.4%)	139 (7.2%)
History of UTI	No	2699 (93.6%)	1708 (88.8%)
	Yes	81 (2.8%)	140 (7.3%)
	Not known	104 (3.6%)	76 (4.0%)
Temperature	<39°C	2780 (96.4%)	1843 (95.8%)
	≥39°C	104 (3.6%)	81 (4.2%)
Urine dipstick	Negative	2511 (87.1%)	1881 (97.8%)
nitrite	Positive	373 (12.9%)	43 (2.2%)
Urine dipstick leukocytes	Negative/trace	2423 (84.0%)	1715 (89.1%)
	Positive	461 (16.0%)	209 (10.8%)
Routine health service laboratory result	Negative	2695 (93.5%)	1862 (96.8%)
	Positive	189 (6.6%)	62 (3.2%)
Research laboratory result	Negative	2833 (98.2%)	1887 (98.1%)
	Positive	51 (1.8%)	37 (1.9%)

A total of 251 (5.2%) and 88 (1.8%) samples were classified UTI positive by health service and research laboratories, respectively. The causative organism distributions were similar between laboratories; in the health service laboratory the causative organisms were: *E*. *coli* 71.7%, other/unknown coliforms 22.3% and *Proteus* spp. 6.0%; in the research laboratory: *E*. *coli* 84.1%, other coliform (*Klebsiella* spp., *Enterobacter* spp., *Serratia* spp., *Citrobacter* spp., *Morganella* spp.) 10.2%, *Proteus* spp. 5.7%. Routine health service laboratory positivity was more common in children aged <3 years (6.6%) than in those aged ≥3 years (3.2%). By contrast, rates of research laboratory positivity were similar in these age groups (1.8% and 1.9%, respectively). Only 64 (1.3%) samples were positive in both laboratories. In 187 (3.9%), the health service laboratory result was positive but research laboratory result negative while in 24 (0.5%) the research laboratory result was positive but health service laboratory result negative (Table 2). In clean catch samples, 104 (4.0%) and 59 (2.3%) samples were classified UTI positive by health service and research laboratories, respectively. In nappy pad samples, 147 (6.7%) were classified as UTI positive in health service laboratories, and 29 (1.3%) samples were classified UTI positive by the research laboratory. The distribution of clinician global illness severity scale for routine heath service and research laboratory UTI positive is shown in S2 Fig. The most common clinical diagnoses in the children who were not UTI positive in the health service and research laboratories, respectively, were ‘upper respiratory tract infection’ (31.0% and 31.3%), ‘viral illness’ (16.6% and 17.7%) and otitis media (10.0% and 9.8%).

**Table 2:** Extent of agreement between health service laboratory (HSL) and research laboratory (RL) results.

Age group and sample collection method	N	HSL-ve, RL-ve	HSL-ve, RL+ve	HSL+ve, RL-ve	HSL+ve, RL+ve	Kappa	95% CI
**Both collection methods**	4808	4533	24	187	64	0.36	(0.29, 0.43)
Clean catch	2619	2501	14	59	45	0.54	(0.45, 0.63)
Nappy pad	2189	2032	10	128	19	0.20	(0.12, 0.28)
**≥3 years**	1924	1852	10	35	27	0.53	(0.41, 0.65)
Clean catch	1861	1792	10	32	27	0.55	(0.43, 0.67)
Nappy pad	63	60	0	3	0	N/A	N/A
**<3 years**	2884	2681	14	152	37	0.29	(0.21, 0.36)
Clean catch	758	709	4	27	18	0.52	(0.37, 0.67)
Nappy pad	2126	1972	10	125	19	0.20	(0.12, 0.28)
**<2 years**	1958	1809	7	121	21	0.23	(0.15, 0.31)
Clean catch	173	155	0	12	6	0.47	(0.23, 0.72)
Nappy pad	1785	1654	7	109	15	0.19	(0.10, 0.27)
**≥2 and <3 years**	926	872	7	31	16	0.44	(0.29, 0.59)
Clean catch	585	554	4	15	12	0.54	(0.36, 0.72)
Nappy pad	341	318	3	16	4	0.27	(0.05, 0.50)

-ve: negative, +ve: positive, HSL: health service laboratory, RL: research laboratory. N/A: cannot compute kappa statistic because no samples were classified as positive by the research laboratory.

Overall agreement between the health service and research laboratories was moderate (kappa = 0.36; 95% CI 0.29, 0.43; Table 2). Agreement was better for clean catch samples (0.54; 0.45, 0.63) than for nappy pads (0.20; 0.12, 0.28). For children aged ≥3 years, too few nappy pad samples were available to allow assessment of reliability. For clean catch samples, reliability was similar in children aged ≥3 years (0.55; 0.43, 0.67) and <3 years (0.52; 0.37, 0.67), which was better than for nappy pad samples in children aged <3 years (0.20; 0.12, 0.28). Similar patterns were seen when comparisons were further stratified into age groups <2 and ≥2 to <3 years, suggesting that lower reliability was attributable to the nappy pad sampling rather than child’s age. Agreement between the health service and research laboratories was low when both leukocyte and nitrite dipstick test results were negative (kappa 0.26 [95% CI 0.12, 0.40] for clean catch samples and 0.05 [-0.02, 0.11] for nappy pad samples, S3 Table).

**Fig 1:**
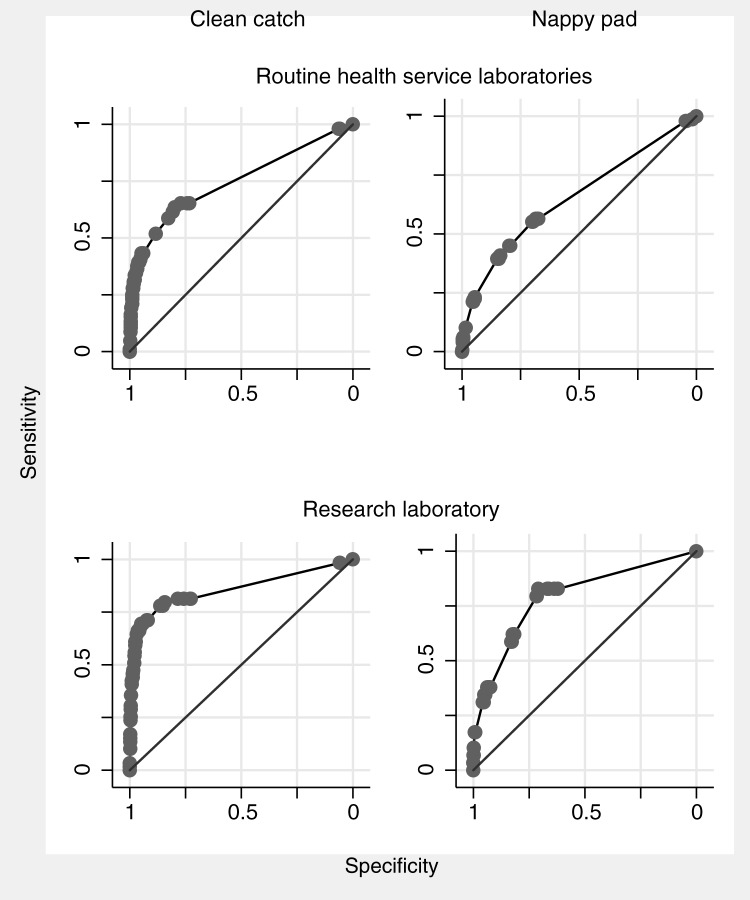
ROC curves from multivariable logistic regression models examining associations of symptoms, signs, and urine dipstick tests with urine culture positivity in routine health service laboratories and the research laboratory.

There was little evidence that passing urine more often or temperature ≥39°C were associated with UTI positivity (Table 3 and S4 Table). Associations of pain or crying when passing urine, and dipstick nitrite and leukocyte positivity, were markedly stronger in clean catch than nappy pad samples and with research laboratory than health service laboratory positivity. Associations with change in urine appearance did not differ markedly between health service and research laboratories. For both clean catch and nappy pad samples, values of the AUC were lower for health service than research laboratories (Table 3 and Fig 1). For clean catch samples the AUC was 0.75 (95% CI 0.69, 0.80) for health service laboratory positivity and 0.86 (0.79, 0.92) for research laboratory positivity. Values of the AUC were markedly lower in nappy pad samples: 0.65 (0.61, 0.70) for health service laboratory positivity and 0.79 (0.70, 0.88) for research laboratory positivity.

**Table 3:** Results from multivariable logistic regression models examining associations of symptoms, signs and urine dipstick tests with separate routine health service and research laboratory results.

	Clean catch		Nappy pad	
	OR (95% CI)	p	OR (95% CI)	p
**Health service laboratories**				
Pain/crying passing urine	2.9 (1.6, 5.1)	<0.001	1.1 (0.4, 3.1)	0.838
Passing urine more often	0.6 (0.3, 1.1)	0.073	0.7 (0.3, 1.5)	0.370
Change in urine appearance	3.0 (1.8, 4.9)	<0.001	2.1 (1.3, 3.5)	0.005
Temperature ≥39°C	1.7 (0.8, 3.8)	0.157	0.7 (0.2, 2.2)	0.526
Dipstick: nitrite +ve	7.6 (4.1, 14.1)	<0.001	2.0 (1.4, 2.9)	0.001
Dipstick: leukocyte +ve	3.1 (1.9, 5.1)	<0.001	3.1 (2.1, 4.4)	<0.001
N observations (N +ve)	2619 (104)		2189 (147)	
AUC (95% CI)	0.75 (0.69, 0.80)		0.65 (0.61, 0.70)	
**Research laboratory**				
Pain/crying passing urine	6.0 (3.0, 11.8)	<0.001	1.4 (0.3, 7.0)	0.716
Passing urine more often	0.8 (0.4, 1.7)	0.543	1.2 (0.3, 4.4)	0.839
Change in urine appearance	3.1 (1.6, 6.1)	0.001	3.1 (1.2, 7.9)	0.019
Temperature ≥39°C	1.7 (0.6, 5.1)	0.333	1.1 (0.1, 8.8)	0.930
Dipstick: nitrite +ve	11.2 (5.4, 23.1)	<0.001	5.2 (2.4, 11.3)	<0.001
Dipstick: leukocyte +ve	5.3 (2.8, 10.0)	<0.001	4.1 (1.9, 8.9)	<0.001
N observations (N +ve)	2619 (59)		2189 (29)	
AUC (95% CI)	0.86 (0.79, 0.92)		0.79 (0.70, 0.88)	

OR: odds ratio, +ve: positive, N: number.

For clean catch samples, the values of the AUC were similar for children aged <3 and ≥3 years, for both health service laboratory and research laboratory positivity (S5 Table). For the research laboratory, but not health service laboratories, AUC values were higher for samples collected in surgery compared with those collected at home. AUC values were similar in samples received by both laboratories within 24 hours compared to samples received after 24 hours, except for nappy pad samples sent to the research laboratory. For both health service and research laboratories the AUC increased with increasing threshold of pure/predominant growth count. For research laboratory positivity, values of the AUC were markedly lower for pure/predominant growth <10^5^ CFU/mL. Values of the AUC were markedly higher in samples with white blood cell count ≥30/mm^3^, except for research laboratory positivity in nappy pad samples. There was little evidence that values of the AUC were higher for research laboratory positivity with pure, compared with predominant, growth.

## Discussion

Based on a large, unselected cohort of children presenting with acute illness to primary care in England and Wales, reliability of microbiological diagnosis of UTI was worse using nappy pad than clean catch samples. The prevalence of microbiological positivity was much higher for health service laboratories than the research laboratory, particularly for nappy pad samples. Associations of microbiological positivity with pre-specified symptoms, signs and urine dipstick test results were lower for health service laboratories than the research laboratory, and for nappy pad than clean catch samples. The reliability of microbiological diagnosis of UTI thus appears better for the research laboratory than for health service laboratories: these results suggest that many of the positive results reported by health service laboratories, particularly those from nappy pad samples, could be due to contamination. Discrimination improved with increasing bacteriuria concentration and pyuria.

To our knowledge, this is the largest and most generalizable primary care-based study comparing the diagnostic performance of health service laboratories with a research laboratory, and using nappy pad and clean catch collection methods. However, our study has limitations, including the potential impact of attrition; 33% of children who were recruited to the DUTY study were not included in this analysis. Children who were not included were younger, highlighting the difficulties of obtaining urine samples from the younger children. The number of UTI positive samples was relatively small, especially for clean catch samples in younger children and for the research laboratory. We minimised asymptomatic bacteriuria by only recruiting children with constitutional or urinary symptoms associated with their acute illness, such that all children found to have significant bacteriuria with a uropathogenic organism would be considered to have a UTI. We minimised selection bias, as where possible we recruited consecutive children; and we asked sites to keep a screening log of patients who were approached but did not take part in the study and the reasons for this. Observers assessing the “index tests” differed from and were blind to the reference standard (and vice versa), thus minimising reviewer bias. A disadvantage of our study design is that we do not know which samples were sent to health service laboratories in containers with boric acid or which ones were refrigerated prior to transport, so were unable to perform exploratory analyses of how these factors may have influenced culture results. Neither were we able to explore the impact of differences between routine health service laboratory procedures and processes. Time to laboratory receipt (within 24 hours or greater than 24 hours) did not appear to influence results. We were not able to obtain a sufficient volume of urine to send a large enough fraction to the research laboratory for all children who submitted a urine sample, as we prioritised the routine health service laboratory fraction in order to ensure that clinicians were sent laboratory results for clinical purposes.

There is not universal agreement on the value of dipstick testing in general,[6] and specifically leukocyturia, in the diagnosis of UTI in children. Furthermore, recommended bacteriuria thresholds differ between laboratory guidelines. European paediatric guidelines suggest a threshold of ≥10^4^ CFU/mL if symptoms are present and ≥10^5^ CFU/mL if no symptoms are present for mid-stream specimens, and lower thresholds for specimens collected by bladder catheterisation or suprapubic aspiration.[9] The UK Standards for Microbiological Investigations do not give specific paediatric guidance but suggest a threshold of a single organism ≥1x10^4^ CFU/mL indicates UTI, though leukocyturia is not required and other thresholds are discussed.[10] US guidance requires both leukocyturia plus a threshold of ≥5x10^4^ CFU/mL.[7] Part of the explanation is that leukocyturia has been identified in children with fever but no UTI.[13] Since study urine samples were processed by UK laboratories, we were obliged to use the UK definition, which does not include leukocyturia. One advantage of this was that it allowed an assessment of the strength of association between leukocyturia and routine health service/research laboratory confirmed UTI, which would not have been statistically valid had leukocyturia been incorporated into laboratories’ definitions. Both leukocyte and nitrite dipstick positivity were associated with microbiologically confirmed UTI in both routine health service and research laboratories, and agreement between health service and research laboratory results was poor when neither dipstick result was positive, which may be because UTI culture positivity is more likely to be due to contamination when dipstick results are negative. Thus, our results support the usefulness of dipsticks as a near patient test in children with suspected UTI.

Microbiological examination of urine requires quantification of bacteria and differentiation of mixed from pure growths. The pour plate method has proved too labour-intensive given the large number of samples submitted to routine microbiology laboratories in the UK: in 2012 663,355 samples (12,689 from children aged <5 years) were submitted in Wales alone, equating to some 12.1 m samples annually (250 000 from children aged <5 years) in England and Wales. The need for rapid throughput led to development of methods using calibrated loops, filter paper strips, or multipoint methods to deliver a standard inoculum.[14–16] All were validated against viable counts performed by pour plates or the method of Miles and Misra.[17] The Standards for Microbiological Investigation followed by most UK laboratories provide options for urine culture using these methods to inoculate CLED or Chromogenic agar: difficulties in defining mixed growths and achieving accurate bacterial counts may be due to small volumes of urine inoculated onto small areas of agar.[14, 18] Spiral plating, which was used by the research laboratory and involves a much larger inoculum (50 μL) over an entire agar plate, quantifies bacterial counts more accurately and allows differentiation of mixed cultures.[19] Further improvements might be achieved through better transport procedures.

Our results suggest that the diagnostic performance of routine UK routine health service laboratory testing may be sub-optimal, and could lead to overtreatment and unnecessary investigations. In adults, results from urine microbiology can be interpreted light of the patient’s presentation. However in young children the difficulties in obtaining uncontaminated samples, together with the non-specific nature of the presenting symptoms, mean there is greater reliance on the laboratory result. More detailed routine microbiological examination of paediatric urine samples could be better justified if urines were selected for testing through an algorithm that increased the prior probability of positivity.[20] Our results suggest that health service laboratories using procedures and processes similar to NHS laboratories should distinguish primary care paediatric (age <5 years) samples from adult samples and consider processing and reporting these using methods akin to our research laboratory, and that national procedures should be correspondingly updated. We did not seek to investigate the impact of differences in the way routine health service laboratories process samples, but further investigation into this could suggest the need for greater harmonisation.

The reliability of agreement of microbiological diagnosis of UTI comparing routine health service laboratories with a research laboratory was moderate for clean catch samples and poor for nappy pad samples and reliability is lower for nappy pad than for clean catch samples. Positive results from the research laboratory appear more likely to reflect real UTIs than those from routine health service laboratories, many of which (particularly from nappy pad samples) appear due to contamination. Health service laboratories should consider merging their procedures and processes towards those used in the research laboratory for paediatric urine samples. Primary care clinicians should try to obtain clean catch samples, even in very young children.

## Supporting information

S1 Fig DUTY study participant flow diagram. (PDF)

S2 Fig Distribution of clinician global illness severity scale. (PDF)

S1 Table Routine health service laboratory Standard Operating Procedures. (PDF)

S2 Table Research laboratory Standard Operating Procedures. (PDF)

S3 Table Extent of agreement between laboratories, by sample collection method and dipstick results. (PDF)

S4 Table Crude results from logistic regression models of symptoms, signs and urine dipstick tests. (PDF)

S5 Table Areas under the ROC curve from logistic regression models. (PDF)

S1 Text Further details of study methods. (PDF)
