# Supplementary material for: Comparison of microbiological diagnosis of urinary tract infection in young children by routine health service laboratories and a research laboratory: Diagnostic cohort study
Source: PLoS One. 2017 Feb 15;12(2):e0171113. doi: 10.1371/journal.pone.0171113 (PMC5310769; doi:10.1371/journal.pone.0171113)
Supplement: S1 Fig — (PDF) [file pone.0171113.s001.pdf]

## S1 Fig. DUTY study participant flow diagram

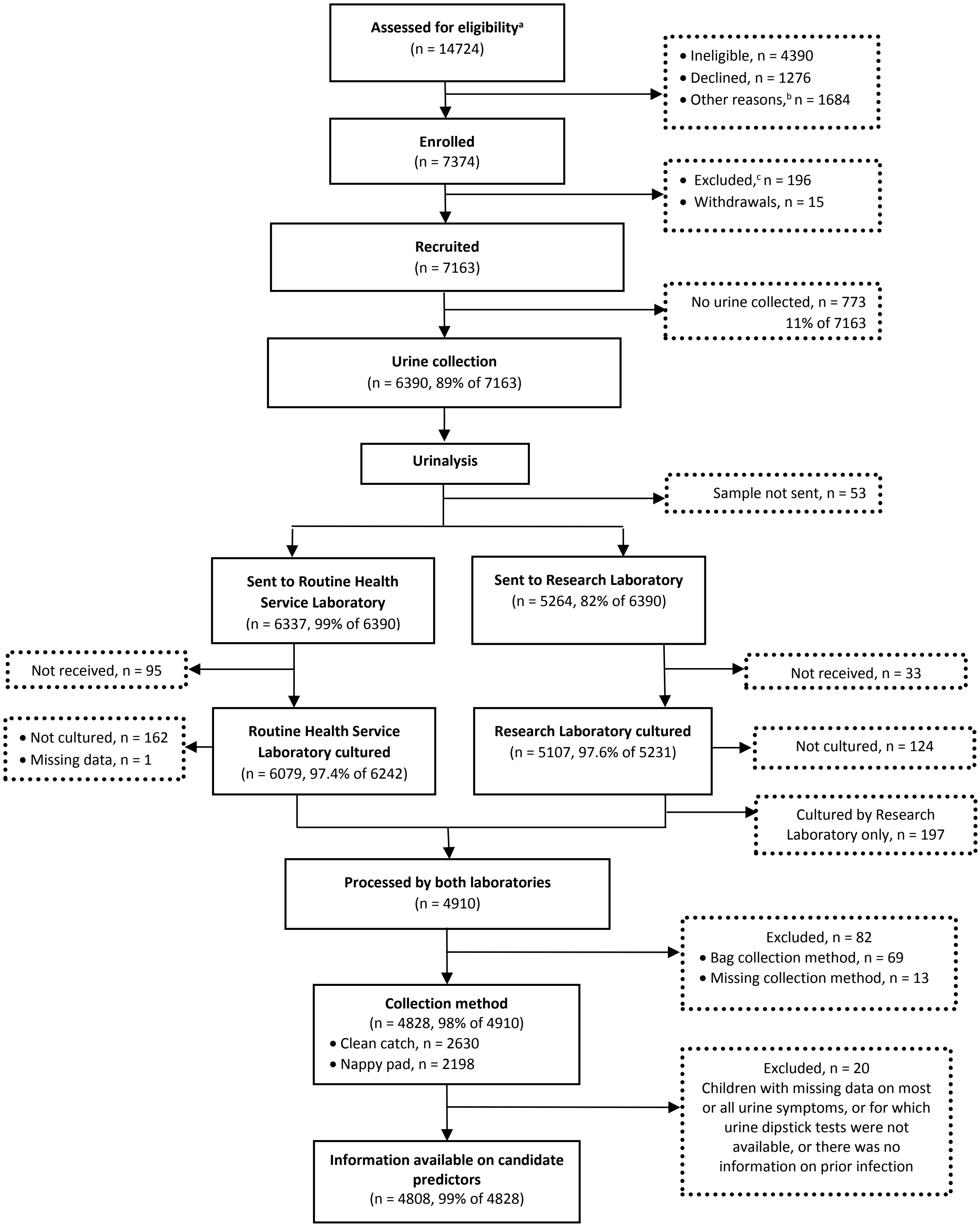


Notes: The original sample size plan was to recruit 6000 children, this was targeted at the DUTY study main objective, namely the development of the clinical algorithm. As this target was met early and the prevalence of UTI was lower than expected, we obtained ethical approval to continue recruiting until April 2012 even though this exceeded the original projected study requirement. Note a, 14,724 children were assessed for eligibility according to completed screening logs. However, this figure is an underestimation of the total number of children assessed, as not all sites completed screening logs and some were incomplete; Note b, other reasons include left prior to invitation, no consent or there was a language barrier; Note c, includes n = 44 retrospectively ineligible due to GP referral, n = 55 data quality issues and n = 97 cases removed during data cleaning.
