# Supplementary material for: Comparison of microbiological diagnosis of urinary tract infection in young children by routine health service laboratories and a research laboratory: Diagnostic cohort study
Source: PLoS One. 2017 Feb 15;12(2):e0171113. doi: 10.1371/journal.pone.0171113 (PMC5310769; doi:10.1371/journal.pone.0171113)
Supplement: S2 Fig — (PDF) [file pone.0171113.s002.pdf]

S2 Fig. Distribution of clinician global illness severity scale

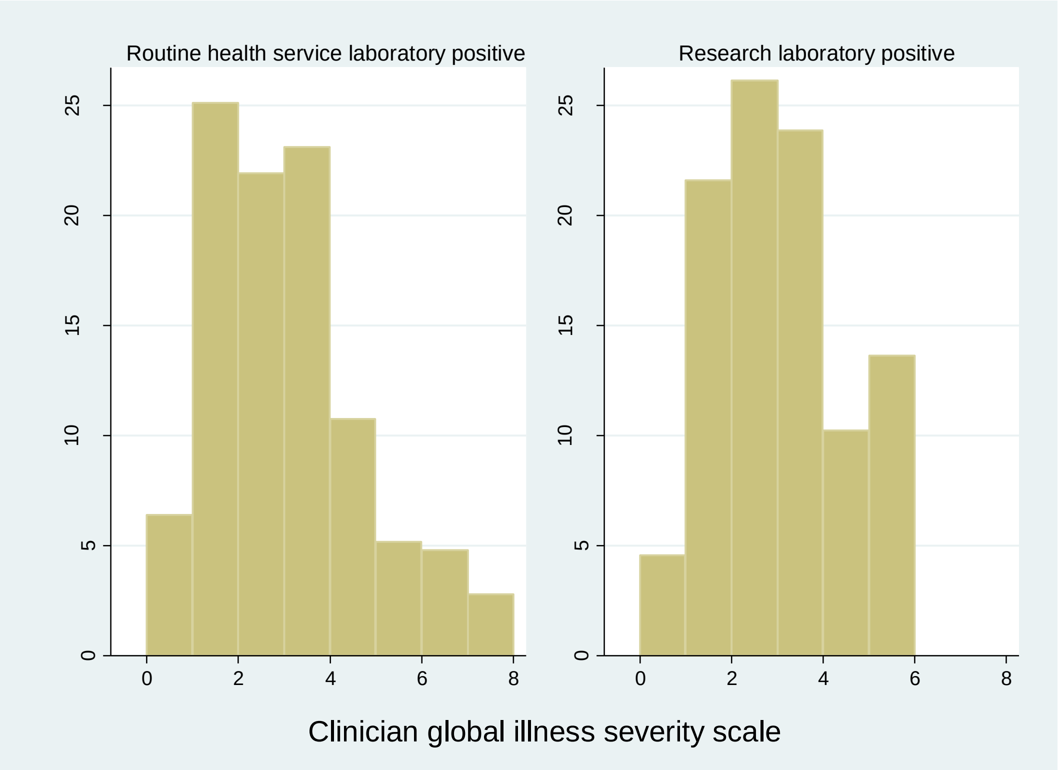

Notes: A score of 0 meant the child was completely well and a score of 10 meant that the child was extremely unwell. The median (interquartile range) of the score was 2 (1-3) for both NHS and research laboratory UTI positive.
