# Supplementary material for: Comparison of microbiological diagnosis of urinary tract infection in young children by routine health service laboratories and a research laboratory: Diagnostic cohort study
Source: PLoS One. 2017 Feb 15;12(2):e0171113. doi: 10.1371/journal.pone.0171113 (PMC5310769; doi:10.1371/journal.pone.0171113)
Supplement: S1 Table — (PDF) [file pone.0171113.s003.pdf]

S1 Table. Routine health service laboratory Standard Operating Procedures

Microscopy: *Standards Unit, Microbiology Services Division, HPA Bacteriology | B 41 | Issue no: 7.1| Issue date: 13.08.12*	Manual	Automated	Dipstix	
Number of laboratories using method	16	8	2*	
Culture Method: *Standards Unit, Microbiology Services Division, HPA Bacteriology | B 41 | Issue no: 7.1| Issue date: 13.08.12*	Paper foot	Calibrated loop	Multipoint	
Number of laboratories using method	4	19	2	
Culture Media: *Standards Unit, Microbiology Services Division, HPA Bacteriology | B 41 | Issue no: 7.1| Issue date: 13.08.12*	CLED	Chromogenic media	Both	
Number of laboratories using media	7	14	4	
Calibrated loop culture volume: *Standards Unit, Microbiology Services Division, HPA Bacteriology | B 41 | Issue no: 7.1| Issue date: 13.08.12*	1uL	2uL	3uL	10uL
Number of laboratories using volume	11	5	2	1
Calibrated loop culture volume: *Standards Unit, Microbiology Services Division, HPA Bacteriology | B 41 | Issue no: 7.1| Issue date: 13.08.12*	1/4	1/3	1/2	Whole
Number of laboratories using plate area	13	2	3	1

* One laboratory uses Dipstix as well as manual microscopy

All participating laboratory methods were a variation on the Standard Microbiology Investigation (SMI) B41 guidelines. Based on 25 NHS laboratory Standard Operating procedures (SOPs) available.
