# Supplementary material for: Comparison of microbiological diagnosis of urinary tract infection in young children by routine health service laboratories and a research laboratory: Diagnostic cohort study
Source: PLoS One. 2017 Feb 15;12(2):e0171113. doi: 10.1371/journal.pone.0171113 (PMC5310769; doi:10.1371/journal.pone.0171113)
Supplement: S2 Table — (PDF) [file pone.0171113.s004.pdf]

S2 Table. Research laboratory Standard Operating Procedures

Microscopy:	Automated
Antimicrobial substance assay	Phenotypic
Culture Method:	Precise colony counts from spiral plater
Culture Media:	
Total count from	Columbia blood agar
Species specific count from	Chromogenic media
Culture volume:	50uL
Culture plate	Whole
