# Supplementary material for: Comparison of microbiological diagnosis of urinary tract infection in young children by routine health service laboratories and a research laboratory: Diagnostic cohort study
Source: PLoS One. 2017 Feb 15;12(2):e0171113. doi: 10.1371/journal.pone.0171113 (PMC5310769; doi:10.1371/journal.pone.0171113)
Supplement: S3 Table — (PDF) [file pone.0171113.s005.pdf]

S3 Table. Extent of agreement between laboratories, by sample collection method and dipstick results

Sample collection method and dipstick results	N	HSL-ve, RL-ve	HSL-ve, RL+ve	HSL+ve, RL-ve	HSL+ve, RL+ve	Kappa	95% CI
**Clean catch**							
leukocyte +ve/nitrite +ve	40	20	4	3	13	0.64	(0.40, 0.88)
leukocyte +ve/nitrite -ve	254	225	3	10	16	0.68	(0.52, 0.84)
leukocyte -ve/nitrite +ve	30	20	0	3	7	0.76	(0.50, 1.00)
leukocyte -ve/nitrite -ve	2295	2236	7	43	9	0.26	(0.12, 0.40)
**Nappy pad**							
leukocyte +ve/nitrite +ve	98	71	2	18	7	0.32	(0.11, 0.53)
leukocyte +ve/nitrite -ve	278	244	1	27	6	0.27	(0.09, 0.45)
leukocyte -ve/nitrite +ve	248	227	3	14	4	0.29	(0.05, 0.53)
leukocyte -ve/nitrite -ve	1565	1490	4	69	2	0.05	(-0.02, 0.11)

-ve: negative, +ve: positive, HSL: health service laboratory, RL: research laboratory
