# Supplementary material for: Comparison of microbiological diagnosis of urinary tract infection in young children by routine health service laboratories and a research laboratory: Diagnostic cohort study
Source: PLoS One. 2017 Feb 15;12(2):e0171113. doi: 10.1371/journal.pone.0171113 (PMC5310769; doi:10.1371/journal.pone.0171113)
Supplement: S4 Table — (PDF) [file pone.0171113.s006.pdf]

S4 Table. Crude results from logistic regression models of symptoms, signs and urine dipstick tests

	**Clean catch**		**Nappy pad**	
	**OR (95% CI)**	**p**	**OR (95% CI)**	**p**
**Health Service laboratories**				
Pain/crying passing urine	5.7 (3.6, 9.1)	<0.001	1.8 (0.7, 4.7)	0.220
Passing urine more often	1.8 (1.1, 2.9)	0.014	1.0 (0.5, 2.0)	0.943
Change in urine appearance	4.6 (3.0, 6.9)	<0.001	2.4 (1.5, 3.8)	<0.001
Temperature ≥39°C	2.0 (1.0, 4.1)	0.056	0.7 (0.2, 2.3)	0.551
Dipstick: nitrite +ve	18.7 (11.0, 32.0)	<0.001	2.4 (1.6, 3.5)	<0.001
Dipstick: leukocyte +ve	6.1 (4.0, 9.2)	<0.001	3.5 (2.5, 5.0)	<0.001
N observations (N +ve)	2619 (104)		2189 (147)	
**Research laboratory**				
Pain/crying passing urine	14.0 (8.1, 23.9)	<0.001	3.7 (0.9, 16.2)	0.079
Passing urine more often	3.6 (2.1, 6.2)	<0.001	2.0 (0.6, 6.6)	0.269
Change in urine appearance	7.4 (4.4, 12.5)	<0.001	4.4 (1.9, 10.1)	<0.001
Temperature ≥39°C	1.9 (0.8, 4.9)	0.173	1.2 (0.2, 9.2)	0.841
Dipstick: nitrite +ve	37.5 (20.7, 68.0)	<0.001	6.8 (3.2, 14.3)	<0.001
Dipstick: leukocyte +ve	14.0 (8.1, 23.9)	<0.001	6.2 (2.9, 12.9)	<0.001
N observations (N +ve)	2619 (59)		2189 (29)	
