# Supplementary material for: Comparison of microbiological diagnosis of urinary tract infection in young children by routine health service laboratories and a research laboratory: Diagnostic cohort study
Source: PLoS One. 2017 Feb 15;12(2):e0171113. doi: 10.1371/journal.pone.0171113 (PMC5310769; doi:10.1371/journal.pone.0171113)
Supplement: S5 Table — (PDF) [file pone.0171113.s007.pdf]

S5 Table. Areas under the ROC curve from logistic regression models

Characteristic of stratified analysis or definition of UTI positivity	Clean catch		Nappy pad	
	N obs (N +ve)	AUC (95% CI)	N obs (N +ve)	AUC (95% CI)
Routine health service laboratory positive, age <3 years	758 (45)	0.75 (0.67, 0.84)	2126 (144)	0.66 (0.61, 0.71)
Routine health service laboratory positive, age ≥3 years	1861 (59)	0.74 (0.67, 0.81)	63 (3)	Too few
Routine health service laboratory positive, age< 3 years, with "not known"±	758 (45)	0.76 (0.68, 0.85)	2126 (144)	0.66 (0.61, 0.71)
Research laboratory positive age <3 years	758 (22)	0.88 (0.80, 0.97)	2126 (29)	0.79 (0.70, 0.88)
Research laboratory positive age ≥3 years	1861 (37)	0.85 (0.77, 0.94)	63 (0)	Too few
Research laboratory positive age< 3 years, with "not known" ±	758 (22)	0.89 (0.81, 0.98)	2126 (29)	0.81 (0.72, 0.89)
Routine health service laboratory positive, surgery sample	2012 (84)	0.73 (0.67, 0.80)	935 (64)	0.66 (0.58, 0.73)
Routine health service laboratory positive, home sample	607 (20)	0.81 (0.71, 0.92)	1254 (83)	0.66 (0.59, 0.72)
Research laboratory positive, surgery sample	2012 (47)	0.90 (0.84, 0.96)	935 (12)	0.84 (0.73, 0.95)†
Research laboratory positive, home sample	607 (12)	0.73 (0.55, 0.90)†	1254 (17)	0.76 (0.64, 0.89)†
Routine health service laboratory positive, sample receipt <24 hours	1959 (76)	0.76 (0.69, 0.82)	1518 (108)	0.67 (0.31, 0.72)
Routine health service laboratory positive, sample receipt ≥24 hours	660 (28)	0.73 (0.64, 0.83)	671 (39)	0.62 (0.53, 0.70)†
Research laboratory positive, sample receipt <24 hours	794 (15)	0.89 (0.77, 1.00)	630 (7)	0.94 (0.87, 1.00)†
Research laboratory positive, sample receipt ≥24 hours	1825 (44)	0.84 (0.76, 0.92)	1559 (22)	0.75 (0.64, 0.86)†
Routine health service laboratory pure/predominant growth $\geq 10^5$ CFU	2619 (104)	0.75 (0.69, 0.80)	2189 (147)	0.65 (0.61, 0.70)
Routine health service laboratory pure/predominant growth $\geq 10^3$-$<10^5$ CFU	2515 (47)	0.58 (0.51, 0.66)†	2042 (40)	0.57 (0.48, 0.65)†
Research laboratory p/p $\geq 10^7$ CFU	2593 (33)	0.89 (0.81, 0.97)	2166 (6)	0.74 (0.60, 0.89)†
Research laboratory p/p $\geq 10^6$-$<10^7$ CFU	2573 (13)	0.84 (0.70, 0.98)	2166 (6)	0.96 (0.92, 1.00)†
Research laboratory p/p $\geq 10^5$-$<10^6$ CFU	2573 (13)	0.79 (0.64, 0.94)	2169 (9)	0.81 (0.68, 0.94)†
Research laboratory p/p $\geq 10^4$-$<10^5$ CFU	2560 (24)	0.59 (0.51, 0.68)†	2160 (61)	0.59 (0.54, 0.64)†
Research laboratory p/p $\geq 10^3$-$<10^4$ CFU	2560 (110)	0.57 (0.52, 0.62)	2160 (93)	0.61 (0.56, 0.66)
Routine health service laboratory positive and WBC# $\geq 30/mm^3$	2572 (57)	0.85 (0.79, 0.91)	2068 (26)	0.74 (0.62, 0.86)
Routine health service laboratory positive and WBC# $<30/mm^3$	2562 (47)	0.63 (0.55, 0.71)	2163 (121)	0.64 (0.59, 0.69)
Research laboratory positive and WBC# $\geq 30/mm^3$	2599 (39)	0.97 (0.93, 1.00)	2164 (4)	0.79 (0.48, 1.00)†
Research laboratory positive and WBC# $<30/mm^3$	2580 (20)	0.67 (0.54, 0.81)†	2185 (25)	0.80 (0.71, 0.89)†
Research laboratory pure growth $\geq 10^5$ CFU	2604 (44)	0.84 (0.76, 0.92)	2172 (12)	0.83 (0.72, 0.94)†
Research laboratory predominant growth $\geq 10^5$ CFU	2575 (15)	0.92 (0.84, 1.00)	2177 (17)	0.76 (0.64, 0.89)†

± Including "not known" responses for "Pain/crying when passing urine" and "Passing urine more often"; †Not all variables included because of perfect prediction; # WBC: white blood cell count.
