# Supplementary material for: Comparison of microbiological diagnosis of urinary tract infection in young children by routine health service laboratories and a research laboratory: Diagnostic cohort study
Source: PLoS One. 2017 Feb 15;12(2):e0171113. doi: 10.1371/journal.pone.0171113 (PMC5310769; doi:10.1371/journal.pone.0171113)
Supplement: S1 Text — (PDF) [file pone.0171113.s008.pdf]

S1 Text. Further details of study methods

Further details of urine collection procedures and laboratory processing

Clean catch samples: This method was used for children who were toilet trained or for whom the parent/guardian was happy to attempt such collection. For toilet trained children we used a small sterile bowl that could fit in a potty or that the parent could hold under the child while s/he was sitting on the toilet. For other children the parent/guardian cleaned the nappy area using water and then sat with the child on their knee with the bowl placed in the perineal area.

Nappy pad samples: With the child lying down, the parent/guardian cleaned the nappy area using water or wipes and placed a clean nappy in situ ready to be fastened. The Research Nurse (RN)/Clinical Studies Officer (CSO), having cleaned her/his hands, opened the nappy pad container and placed the pad in the child's perineal area, with minimal handling. The clean nappy was then fastened. Nappy pads were removed as soon as the child urinated, to reduce the risk of contamination. If the child had not passed urine after 30 minutes, the perineum was re-cleaned and a fresh pad was inserted. The nappy area was re-cleaned and a clean pad was re-inserted if the pad became contaminated with faeces.

Urine collection bags were used for 100 children: these data were excluded from the present study.

If it was not possible to obtain a sample before the child left the primary care site, the parent/guardian was given the necessary equipment and advice on taking the sample at home. They were given a labelled Sterilin™ bottle into which to transfer the urine, and asked to write the time and date the sample was obtained. They were advised to refrigerate the sample and return it to the primary care site as soon as possible, preferably within 24 hours. The RN/CSO telephoned parents/guardians the next day to remind them to return the sample and, where feasible, offered to collect the sample from the child's home.

A minimum of 1ml urine volume was required for processing by each laboratory.

Further details of data analyses

A small number of samples were excluded: those for which there were missing data on most or all urine symptoms (5 samples), or for which urine dipstick tests were not available (12 samples), or

there was missing information on prior infection (3 samples). Missing data on temperature (204 children) were coded as <39°C. The remaining, sporadic missing values (on 5 children) were coded as “no or slight problem”.

Additional symptoms and signs thought to be relevant to UTIs mainly for older children were: daytime or bed wetting when previously dry and a history of UTI. These were recorded in too few of the children who provided nappy pad samples to permit their associations with microbiology results to be examined. For children who provided a clean catch sample, we confirmed that associations of these variables with UTI were sufficiently modest that the AUC were little changed when they were excluded from the logistic regression models (results available from the authors). Models were therefore restricted to the remaining six symptoms, signs and dipstick test results, for both clean catch and nappy pad samples.
